# A comparative molecular survey of malaria prevalence among Eastern chimpanzee populations in Issa Valley (Tanzania) and Kalinzu (Uganda)

**DOI:** 10.1186/s12936-016-1476-2

**Published:** 2016-08-19

**Authors:** Mwanahamisi I. Mapua, Klára J. Petrželková, Jade Burgunder, Eva Dadáková, Kristýna Brožová, Kristýna Hrazdilová, Fiona A. Stewart, Alex K. Piel, Peter Vallo, Hans-Peter Fuehrer, Chie Hashimoto, David Modrý, Moneeb A. Qablan

**Affiliations:** 1 Department of Pathology and Parasitology, University of Veterinary and Pharmaceutical Sciences Brno, 612 42 Brno, Czech Republic; 2 Institute of Vertebrate Biology, Czech Academy of Sciences, 603 00 Brno, Czech Republic; 3 Liberec Zoo, 460 01 Liberec, Czech Republic; 4 Institute of Parasitology, Biology Centre, Czech of the Academy of Sciences, 370 05 České Budějovice, Czech Republic; 5 Faculty of Science, Masaryk University, 611 37 Brno, Czech Republic; 6 Department of Infectious Diseases and Microbiology, Faculty of Veterinary Medicine, University of Veterinary and Pharmaceutical Sciences Brno, 612 42 Brno, Czech Republic; 7 Department of Virology, Veterinary Research Institute, 621 00 Brno, Czech Republic; 8 Division of Biological Anthropology, Department of Archaeology and Anthropology, University of Cambridge, Cambridge, CB2 3QG UK; 9 School of Natural Sciences and Psychology, Liverpool John Moores University, Liverpool, L33AF UK; 10 Institute of Evolutionary Ecology and Conservation Genomics, Ulm University, Albert-Einstein Allee 11, 89069 Ulm, Germany; 11 Institute of Parasitology, Department of Pathobiology, University of Veterinary Medicine Vienna, Veterinaerplatz 1, 1210 Vienna, Austria; 12 Primate Research Institute, Kyoto University, Kanrin, Inuyama, Aichi 484-8506 Japan; 13 CEITEC-Central European Institute of Technology, University of Veterinary and Pharmaceutical Sciences Brno, 612 42 Brno, Czech Republic; 14 Department of Veterinary Medicine, College of Food and Agriculture, United Arab Emirates University, PO Box 15551, Al Ain, United Arab Emirates

**Keywords:** Malaria, *Pan troglodytes schweinfurthii*, *Plasmodium* spp., *Laverania*, *Cyt*-*b* gene

## Abstract

**Background:**

Habitat types can affect vector and pathogen distribution and transmission dynamics. The prevalence and genetic diversity of *Plasmodium* spp. in two eastern chimpanzee populations—Kalinzu Forest Reserve, Uganda and Issa Valley, Tanzania—inhabiting different habitat types was investigated. As a follow up study the effect of host sex and age on infections patterns in Kalinzu Forest Reserve chimpanzees was determined.

**Methods:**

Molecular methods were employed to detect *Plasmodium* DNA from faecal samples collected from savanna-woodland (Issa Valley) and forest (Kalinzu Forest Reserve) chimpanzee populations.

**Results:**

Based on a *Cytochrome*-*b* PCR assay, 32 out of 160 Kalinzu chimpanzee faecal samples were positive for *Plasmodium* DNA, whilst no positive sample was detected in 171 Issa Valley chimpanzee faecal samples. Sequence analysis revealed that previously known *Laverania* species (*Plasmodium reichenowi*, *Plasmodium billbrayi* and *Plasmodium billcollinsi*) are circulating in the Kalinzu chimpanzees. A significantly higher proportion of young individuals were tested positive for infections, and switching of *Plasmodium* spp. was reported in one individual. Amongst the positive individuals sampled more than once, the success of amplification of *Plasmodium* DNA from faeces varied over sampling time.

**Conclusion:**

The study showed marked differences in the prevalence of malaria parasites among free ranging chimpanzee populations living in different habitats. In addition, a clear pattern of *Plasmodium* infections with respect to host age was found. The results presented in this study contribute to understanding the ecological aspects underlying the malaria infections in the wild. Nevertheless, integrative long-term studies on vector abundance, *Plasmodium* diversity during different seasons between sites would provide more insight on the occurrence, distribution and ecology of these pathogens.

**Electronic supplementary material:**

The online version of this article (doi:10.1186/s12936-016-1476-2) contains supplementary material, which is available to authorized users.

## Background

Parasite distribution and transmission dynamics are influenced by the ecological context of the host-parasite interactions and a variety of local environmental parameters [1–3]. In the case of vector-borne *Plasmodium* infections, the primary effect of habitat on the transmission of malaria is by affecting larvae development, abundance and distribution of competent vectors [4–7]. Numerous studies have demonstrated the relationship between specific habitats and levels of *Plasmodium* infections in humans [8–12]. However, research addressing habitat types as a source of variation in prevalence and diversity of these parasites in wild apes is lacking. In addition to habitat, host traits such as age, sex and host density may also have an influence on host parasite infection and transmission of *Plasmodium* spp. [13–15].

Chimpanzees (*Pan troglodytes*), like several other primates, harbour a multitude of malaria parasites. With the development and refinement of molecular diagnostic techniques together with non-invasive sampling, at least seven distinct *Plasmodium* species are known to infect wild chimpanzees. Four of them, *Plasmodium reichenowi*, *Plasmodium gaboni*, *Plasmodium billcollinsi* and *Plasmodium billbrayi* belong to the subgenus *Laverania* and are chimpanzee-host specific [16–22]. The remaining three species, usually referred to as *Plasmodium malariae*-like, *Plasmodium ovale*-like and *Plasmodium vivax*-like, rarely occur in chimpanzees and they are genetically related to their human counterparts. Nevertheless, the nomenclature of these rare taxa requires further investigation [19]. Given the high genetic diversity of *Plasmodium* species reported from chimpanzees and other primates including humans [19, 20, 23], a better understanding of the infection dynamics and interactions between parasites, *Anopheles* mosquitoes, hosts and environmental parameters that facilitate malaria transmission in apes is required [15, 18, 24].

In the current study, the prevalence and genetic diversity of *Plasmodium* spp. was investigated in two populations of eastern chimpanzees (*P. t. schweinfurthii*) inhabiting two different habitats: (1) savanna woodlands in Issa Valley, Tanzania and (2) evergreen moist forest in Kalinzu Forest Reserve (KFR), Uganda. Malaria infection was compared between these two habitats (savanna and moist evergreen forest) because of their variable environmental parameters that may influence the exposure to malaria parasites with varying degrees in chimpanzee populations. Because chimpanzees at KFR are habituated, the relationship between age, sex and malaria infection patterns in this population was additionally addressed.

## Methods

### Study sites

#### Issa Valley, Tanzania

**Fig. 1:**
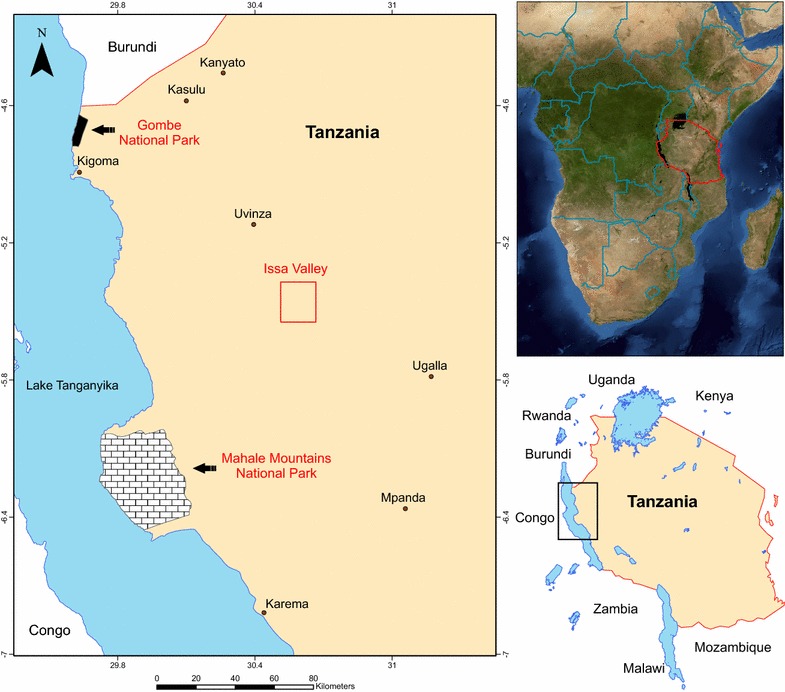
Ugalla Map. Map of the study site Issa Valley, Western Tanzania

The Issa valley is located in western Tanzania (Fig. 1), about 90 km east of Mahale Mountains National Park, and approximately 70 km from Uvinza, the nearest legitimate village. Issa valley is characterised as an open area with no formal protective status, where small-scale illegal human activity for hunting and logging takes place [25]. The entire region is one of the driest and most open chimpanzee habitats, with an altitudinal range of 900–1800 m above sea level [26]. There is an extended dry season (May–September), with rains from October–April, peaking in January (unpublished data), averaging 1095 mm/year (range 835–1395 mm/year). Average daily temperature varies from 11–35 °C [27]. The habitat is dominated by savanna (Miombo) woodland, characterized by *Brachystegia* and *Julbernardia* trees, with small riparian forest patches [26]. The population density of Issa chimpanzees is estimated to be ~0.25 individuals/km^2^ [25]. Data on the prevalence of *P. vivax* in this population have been reported elsewhere [28]. In addition to chimpanzees, several other primate species inhabit the study site, including red colobus monkeys (*Piliocolobus tephrosceles*), yellow baboons (*Papio cynocephalus*), blue (*Cercopithecus mitis*) and red-tailed monkeys (*Cercopithecus ascanius*), vervet monkeys (*Chlorocebus pygerythrus*), bushbabies (*Galago senegalensis*, *Cercopithecus moholi*) and greater galagos (*Otolemur crassicaudatus*) [26].

#### KFR, Uganda

**Fig. 2:**
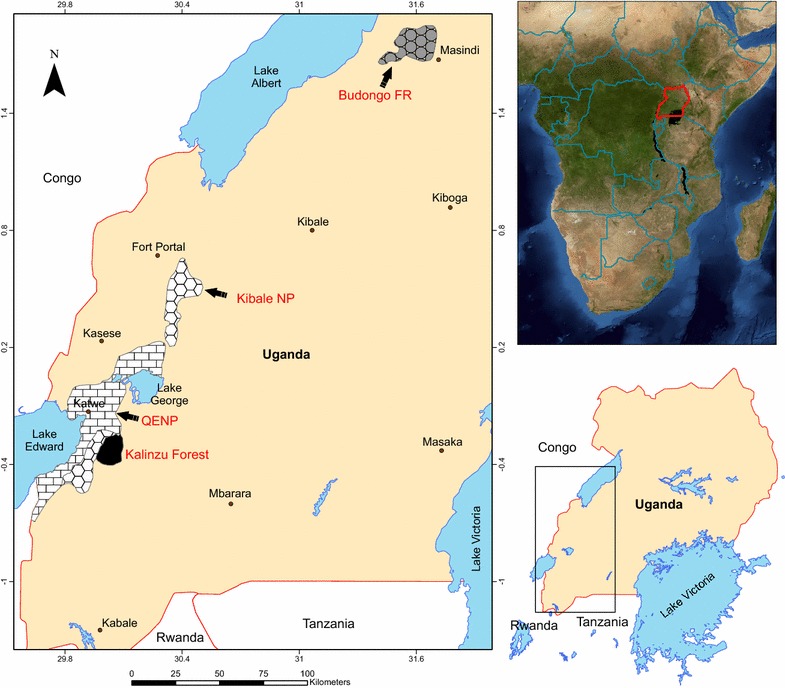
Kalinzu Map. Map of the study site in Kalinzu Forest Reserve, Western Uganda

Kalinzu is one of the three largest forest blocks in Uganda. The forest reserve (~137 km^2^) is located on the eastern ridge of the western Rift valley in western Uganda (Fig. 2), with an altitudinal range of 1200–1500 m above sea level [29]. The area is adjacent to Kashoha–Kitomi Forest Reserve and Maramagambo Forest Reserve on the north and west sides, agricultural fields to the east and tea plantations to the south [29]. Kalinzu has a bimodal distribution of rainfall with peaks between September–December and March–May, and average annual rainfall of 1584 mm. The average daily temperature varies from 15 to 25 °C [30, 31]. The vegetation is classified as medium altitude moist evergreen forest, with common species including *Musangaleo* and *Ficus* spp. [32]. The chimpanzee population density is estimated to be ~1.67 individuals/km^2^ [33]. In addition to *Pan troglodytes schweinfurthii*, black and white colobus (*Colobus guereza*), olive baboons (*Papio anubis*), red tailed (*Cercopithecus ascanius*), blue (*C. mitis*), and L’hoests monkeys (*C. lhoesti*) occur in the area [32].

### Sample collection

#### Issa Valley

171 faecal samples were collected from a single community of chimpanzees inhabiting the Issa study area between March–May 2012 and June–August 2013. It was not possible to attribute the faecal samples to specific individuals. Most of the faecal samples were collected underneath fresh nests (~12 h old) and some from chimpanzee trails. Approximately 20 g of faecal material was collected in a 50 ml tube, containing 20 ml of RNA*later*™ (Ambion Inc., Austin, TX). All faecal samples were stored in a freezer at −20 °C on site, and subsequently shipped to the Czech Republic, where they were kept at −20/−80 °C until DNA extraction.

#### KFR

Between April and July 2014, faecal samples collected from 41 habituated chimpanzees (males, n = 20; females, n = 21). A total of 123 fresh faecal samples, ranging from 1 to 10 faecal samples per individual were collected. Samples were collected during direct observations of chimpanzees. Concurrently, during tracking of chimpanzees 37 faecal samples were collected from unidentified individuals. Collection and storage protocols were the same as those at Issa, with the exception that samples were kept at 4 °C in a fridge at base camp prior to shipping to the Czech Republic, where they were kept at −20/−80 °C until DNA extraction.

### Molecular methods

Total DNA was extracted from 1.5 ml of the faecal—RNA*later*™ suspension using a QIAamp Stool DNA Mini kit (Qiagen, Valencia, CA, USA) and PSP® Spin Stool DNA Kit (Stratec Molecular, Germany) according to the manufacturer’s protocol. Bound DNA was eluted in 100 μl elution buffer. To determine the concentration of the extracted DNA, total DNA was measured by fluorometry, using a Qubit (Invitrogen, Carlsbad, CA, USA). To screen samples for *Plasmodium*, a nested PCR was performed on each sample targeting a ~930 bp fragment of the *Plasmodium cytochrome b* (*cyt*-*b*) gene, as described by Prugnolle et al. [34], with modification of the second PCR reaction. A pair of short internal primers amplifying overlapping fragments (516 and 558 bp) was designed, retrieved sequences were contiged to obtain same region of *cyt*-*b*. First round PCRs were performed in a 25 μl reaction, containing 12.5 μl of PCR mix (Qiagen), 2.5 μl of solution Q (Qiagen) and 0.2 μl of each primer (DW2 and DW4) in 10 pmol concentration and 4 μl of the DNA sample. Second nested PCR was performed using two different set of reactions, using Cytb1 (5′-CTCTATTAATTTAGTTAAAGCACA-3′) and Cytb2B (5′-GCTCTATCATACCCTAAAGG-3′) in the first set, and Cytb2 (5′-ACAGAATAATCTCTAGCACC-3′) and Cytb1A (5′-CAAATGAGTTATTGGGGTGCAACT-3′) for the second set. Two µl of first round PCR product was then used in a second round 25 µl nested PCR reaction, containing 12.5 µl common Master Mix (Top-Bio, Czech Republic) and 1 µl of each primer in 10 pmol concentration. For details of the modified nested PCR conditions see [15]. PCR products were visualized on 2 % agarose gel and stained with Gold-View. Bands of the expected size were visualized using an UV light source, excised, purified using QIAquick gel extraction kit (Qiagen, Germany) and sequenced in both directions using internal primers by Macrogen capillary sequencing services (Macrogen Europe, the Netherlands).

### Sequence and phylogenetic analyses

Sequences were edited in Chromas Pro 1.5 software (Technelysium, Ltd) and alignment was prepared with ClustalW multiple alignment tool implemented in Bioedit Sequence Alignment Editor v.7.0.9.1 [35]. All suitable retrieved sequences were submitted to GenBank™ database under the Accession Numbers KT864824–KT864842.

The alignment was checked manually and the resulting sequence were (~758 bp) later used for phylogenetic analyses. To examine the phylogenetic relationship of the new dataset, sequences from different ape *Plasmodium* species downloaded from GenBank™ were added to the final alignment. For the final analyses, only haplotypes were further included (haplotypes and redundant sequences are shown in Table 1).

**Table 1:** List of haplotypes used in phylogenetic analyses

Haplotype	Isolate	References
KFR144	KFR 144, KFR177, KFR5A, KFR9A, KFR 21, KFR45	This study
	HM235389_Pts, HM235389_Pts	Liu et al. [18]
HM235394	HM235394_Pts	Liu et al. [18]
HM235048	HM235048_Pts	Liu et al. [18]
HM235391	HM235391_Pts, HM235388 _Pts	Liu et al. [18]
HM235029	HM235029_Ptt	Liu et al. [18]
HM235028	HM235028_Ptt	Liu et al. [18]
HM235328	HM235364_Pte, HM235328_Ptt, HM235359_Ptt, HM235299_Ptt	Liu et al. [18]
HM235362	HM235362_Pte, HM235097_Pte, HM235096_Pte, HM235089_Pte	Liu et al. [18]
KFR3A	KFR3A	This study
KFR150	KFR150, KFR167,	This study
	HM235402_Pts, HM235401_Pts	Liu et al. [18]
KFR72	KFR72	This study
HM235341	KFR149	This study
	HM235341_Ptt, HM235339_Ptt, HM235108_Pts, HM235340_Ptt, HM235392_Pts, HM235342_Ptt, HM235395_Pts	Liu et al. [18]
HM235351	HM235351_Ptt	Liu et al. [18]
HM235380	HM235380_Ggg	Liu et al. [18]
HM235367	HM235367_Ggg	Liu et al. [18]
KC175316	KC175316	Sundararaman et al. [49]
AY282929	AY282929	Joy et al. [50]
HM235382	HM23538_Ggg, HM235294 Ggg, HM235304 Ggg	Liu et al. [18]
HM235400	HM235400_Pts, HM235076_Pts, HM235399_Pts	Liu et al. [18]
KFR178	KFR178	This study
HM235320	HM235320	Liu et al. [18]
HM235052	HM235052	Liu et al. [18]
GQ355470	GQ355470_Pts	Krief et al. [17]
GQ355471	GQ355471_Pts	Krief et al. [17]
KFR90	KFR90	This study
KFR36	KFR36	This study
KFR105	KFR105	This study
KFR32A	KFR32A, KFR93, KFR188, KFR7A	This study
FJ895308	FJ895308_Ptt	Ollomo et al. [16]
JX893151	JX893151_Ptt	Pacheco et al. [21]
HM235102	HM235102_Pte	Liu et al. [18]
HM234997Ptt	HM234997_Ptt, HM235315_Ptt, HM235348_Ptt, HM235309_Ptt, HM235280_Ptt	Liu et al. [18]
	HM235114_Pte, HM235113_Ptt, HM235112_Ptt, HM235088_Pte, HM235086_Pte	
	HM235083_Pte	
HM235100	HM235100_Pte	Liu et al. [18]
HM235077	HM235077_Ptt	Liu et al. [18]
HM235375	HM235375_Ggg, HM235284_Ggg	Liu et al. [18]
HM235313	HM235313_Ggg	Liu et al. [18]
JQ240419	JQ240419	Miao et al. [51]
KC175307	KC175307	Sundararaman et al. [49]
AB489194	AB489194	Hayakawa et al. [52]

Acronyms under accession number represent chimpanzee and gorilla sub-species

Ptt *Pan troglodytes troglodytes*, Pte *Pan troglodytes ellioti*, Pts *Pan troglodytes schweinfurthii*, Ggg; *Gorilla gorilla gorilla*

*KFR* Kalinzu forest reserve

Ref. [49]

Ref. [50]

Ref. [51]

Ref. [52]

Phylogenetic relationships were inferred using the maximum likelihood (ML) method under the general time-reversible evolutionary model with gamma distributed substitution rates (GTR + Γ) in program PhyML 3.0 [36]. Nodal support was assessed by bootstrap using 1000 pseudoreplicates. Additionally, Bayesian methods using the program MrBayes 3.2.2 [37] was also used to reconstruct phylogenetic relationships. Setting for the evolutionary model was the same as in ML and the search was carried out in two simultaneous runs of one million generations, sampled each 100 generations, with a burn-in of 25 %.

### Cloning of mixed infection samples

Two samples were cloned separately with a TOPO® TA cloning kit (Invitrogen, Carlsbad, CA, USA) according to the manufacturer’s instructions. Plasmids containing inserts were isolated from positive *Escherichia coli* colonies by GenElute™ plasmid mini prep kit (Sigma-Aldrich, St. Louis, MO, USA). DNA extracts from at least six randomly selected colonies were sequenced in both directions.

### Statistical analyses

Prevalence was defined as the number of *Plasmodium*-positive individuals divided by the total of individuals tested. Samples collected from unidentified individuals were not included for the calculation of prevalence, but they were used to investigate the genetic diversity of the parasites. Of the 41 habituated individuals sampled in KFR, 25 were re-sampled to observe the fluctuation of the infections. In order to examine the possible effect of sex and age on the occurrence of malaria in KFR chimpanzees, a general linear mixed model (GLMM) with binomial distribution was fitted. Since there were a limited number of faecal samples from juveniles and subadults, age classes were pooled and grouped as juveniles/subadults and adults. Age-classes were verified based on previously suggested categorization [38]. Samples were classified according to sex (fixed factor: male, female) and class of age (fixed factor: juvenile/subadult, adult). Individual identity was treated as a random factor. Statistical analyses were performed in R [39].

## Results

In total, 331 chimpanzee faecal samples (Table 2) from Issa Valley and KFR were examined. All faecal samples collected from Issa chimpanzees were negative for *Plasmodium* DNA. On the contrary, *Plasmodium* spp. was detected in 32 out of 160 (both identified and unidentified individuals) faecal samples collected from KFR chimpanzees. In total, 22 out of 123 samples collected from identified individuals were positive for *Plasmodium* DNA;10 out of 37 samples from unidentified individuals were *Plasmodium*-positive. The prevalence among identified individuals was 43.9 % (n = 18/41). The general linear mixed model showed that sex had no significant effect on the susceptibility to infection (GLMM: *z* = −0.027, *p* = 0.283), while age was a significant factor influencing *Plasmodium* infection. The total prevalence of *Plasmodium* spp. was significantly higher among juvenile/subadult individuals than adults (GLMM: *z* = 2.308, *p* = 0.020). Of the re-sampled individuals (n = 25), eleven were found positive at least once. Variation on detection of *Plasmodium* DNA (negative-to-positive and vice versa) was common and observed in 18 identified individuals (Table 3). Switching of *Plasmodium* spp. was observed in one individual (Table 3).

**Table 2:** Results of PCR detection of *Plasmodium* DNA in faeces of chimpanzees from Ugalla and Kalinzu study sites and determination of *Plasmodium* spp. by subsequent sequencing

Field site	*Plasmodium* spp.			
	*P. reichenowi*	*P. billbrayi*	*P. billcollinsi*	Mixed infection
Ugalla (n = 171)	–	–	–	–
Kalinzu (n = 160)	12	11	7	2

**Table 3:** Pattern of *Plasmodium* spp. infection among identified chimpanzees’ individuals

			Sampling time and *Plasmodium* spp. identified			
Individuals	Sex	Age category	April	May	June	July
Buru	M	2	−	−		−
Ross	M	1	−/−	−/−	−	−
Ota	M	1	−		*P. reichenowi*	
Tange	M	2	−/−	−/−	−/−	−
Yawara	M	2	*P. billbrayi*	*P. billcollinsi*/−/−	−/*P. billbrayi*/−/−	
Ichiro	M	2	*P. reichenowi*/-	−/−/−	−	−
Goku	M	2	−	−	−/−/−/−/−	−
Black	M	1	−/−	−/−/*P. billbrayi*	−/−/−/−	−
Gure	M	2	−/−/−	−/−		*P. reichenowi*
Ponta	M	2	−	/−/−/−	*P. billcollinsi*	
Deo	M	2	−	−		−
Pieten	M	1	−/*P. reichenowi*, *P*. *billbrayi*, *P. billcollinsi*		−	−
Kanta	M	1	*P. billcollinsi*	*P. billcollinsi*		
Marute	M		−	−		−
Ricky	M	1	*P. billcollinsi*			
JO	M	1				*P. billbrayi*/*P. billbrayi*
Taike	M	1		*P. billbrayi*	−/−	
Iso	M	1		−	−	
Prince	M	1			−/−/−	
Max	M	1	*P. billbrayi*			
Pinka	F	2	−/−	−		
Kakumu	F	2	−/−			
Tae’s daughter	F	1	−			
Nono	F	2	−	−	−	
Haro	F	2	−	−		
Haruka	F	1	*P. reichenowi*			
Shoko	F	2	−/−		−	−
Tae	F	2	−			
Gai	F	2	*P. billcollinsi*	−		
Migi	F	2		−	−	
Ida	F	2	−			
Iku	F	1	*P. billbrayi*			
Nakko	F	2		*P. reichenowi*		
Kanna	F	2	*P. reichenowi*			
Minny	F	2		−		
Umuoge	F	1		−		
Ume	F	2		−		
Miki	F	1			−	
Rina	F	2			−	
Michio	F	2			−	
Mami	F	2				*P. reichenowi*

− negative for *Plasmodium*, *1* juvenile/sub-adult, *2* adult

Alignment and phylogenetic analysis of the obtained *cyt*-*b* sequences (both from identified and unidentified individuals) with reference sequences indicated the presence of *Plasmodium* strains that specifically infect only chimpanzees (see Additional file 1). Among the retrieved sequences, 12 were *P. reichenowi*, 11 *P. billbrayi* and seven *P. billcollinsi*. All sequences obtained in this study clustered with their homologous sequences retrieved from GenBank™ and form well-supported clades. Geographical sub-structuring among *P. reichenowi* was observed, whereby sequences obtained from *P. t. schweinfurthii* clustered separately from other *P. reichenowi* sequences from *P. t. troglodytes* and *Pan troglodytes ellioti*. No samples containing *cyt*-*b* of *P. gaboni* or non-*Laverania* species (*P. vivax*-like, *P. malariae*-like and *P. ovale*-like) were detected in this dataset. Mixed infections were detected in two samples. Sequences of two PCR amplicons showed double peaks in the chromatograms, suggesting mixed infections.

These samples were further processed by cloning to identify *Plasmodium* to species level. In the first sample (from an unidentified individual), 15 sequences were obtained with two representative sequence patterns that were in agreement with BLAST-searches for the *cyt*-*b* sequences: 14 sequences were 99–100 % similar to *P. reichenowi* (acc. number: HM235389), and one sequence was 99 % similar to *P. billbrayi* (acc. GQ355468). In the second sample (from an identified individual), 12 sequences were obtained with three representative sequences patterns: four sequences were 99 % similar to *P. reichenowi* (acc. number: HM235389), five sequences were 99–100 % to *P. billbrayi* (acc. number: GQ355468), and three sequences were 99 % similar to *P. billcollinsi* (acc. number: HM235392).

## Discussion

A number of studies have described the distribution and genetic diversity of *Plasmodium* spp. in African great apes [17, 18, 22, 34, 40, 41], yet there is substantial lack of knowledge on the effect of intrinsic and extrinsic factors that govern malaria parasite transmission and frequencies of infections in free ranging chimpanzees. This is the first study to investigate the prevalence and genetic diversity of *Plasmodium* spp. in KFR. The findings from KFR are comparable to previous studies by Liu et al. [18], that were conducted at multiple field sites, as well as to the study by Kaiser et al. [41] from Budongo Forest in Uganda. While no *Plasmodium* spp was detected from Issa Valley samples, results from a previous study [28] revealed that four out of three hundred thirteen chimpanzee samples from this population to be positive for *P. vivax*-like. Variation in the prevalence between this study and that of Liu et al. [28] is most likely to be attributable to smaller sample set, and, possibly also to differences in sensitivity of detection methods. Looking at this discrepancy from a different perspective, *P. vivax* tends to stay dormant in the liver for many years [42]. Consequently, it can be speculated that during sampling time shedding of *Plasmodium* DNA into the intestinal lumen was minimal, leading to failure to detect *P. vivax* DNA in faecal samples.

An overall prevalence of *Plasmodium* spp. in KFR was 43.9 %, while all faecal samples from Issa Valley were negative. The remarkable ecological differences between KFR and Issa Valley habitats represent most plausible explanation for observed differences, as they may impact on the species diversity and abundance of anopheline mosquitoes. However, also host density may have significant impact on the transmission and maintenance of infections in a given population [12]. Kalinzu chimpanzees live at a relatively high density (~1.67 individuals/km^2^, [33]) compared to Issa chimpanzees (~0.25 individuals/km^2^, [25]). Then, the abundance of hosts may act as an additional factor influencing the prevalence of *Plasmodium* spp.

Liu et al. [28] screened another but forest-inhabiting eastern chimpanzee population (*Pan t. schweinfurthii*) from Gombe National Park, and none of the samples was positive for *P. vivax*-like. The absence (or very low prevalence Liu et al. [28]) of *Plasmodium* infection is these eastern chimpanzee populations (Issa Valley and Gombe National Park) could be also attributed to the genetic factors related to hosts as observed in human [43] rather than to their habitat. Unfortunately, it is difficult to reliably compare the results of these two studies due to the different diagnostic techniques employed (*P. vivax* species-specific assay in the Gombe study [28], and *Plasmodium* genus-specific in the present study). Nevertheless, screening of near-by forested (Mahale Mountains National Park) and other savanna-dwelling chimpanzees (e.g. Semliki, Uganda; Fongoli, Senegal), as well as re-screening of the Gombe chimpanzee population for presence of *Laverania* species would offer an insight into the factors the influence the occurrence of *Plasmodium* spp. in eastern chimpanzees.

Over the past 5 years, numerous *Plasmodium* species have been reported to circulate in free-ranging great apes [19]. Consistent with previous studies [18, 22, 34, 41], sequence analyses of the *cyt*-*b* gene of *Plasmodium* spp. from Kalinzu chimpanzees revealed a high diversity of malaria parasites. With the exception of *P. gaboni*, which was not detected in this sample set, most of the sequences were identified as *P. billbrayi*, however, *P. reichenowi* and *P. billcollinsi* were also confirmed. Phylogenetic analysis showed that all sequences in the present study cluster within the clades of subgenus *Laverania*, no sequence belonging to non-*Laverania* (*P. vivax*-like, *P. ovale*-like and *P. malariae*-like) lineage was identified. These results agree with recent findings on ape malaria, where *Laverania* lineages were the only ones reported from central chimpanzees across multiple field sites in Gabon [22], although, non-*Laverania* parasites are known to circulate within the same chimpanzee populations [44].

**Fig. 3:**
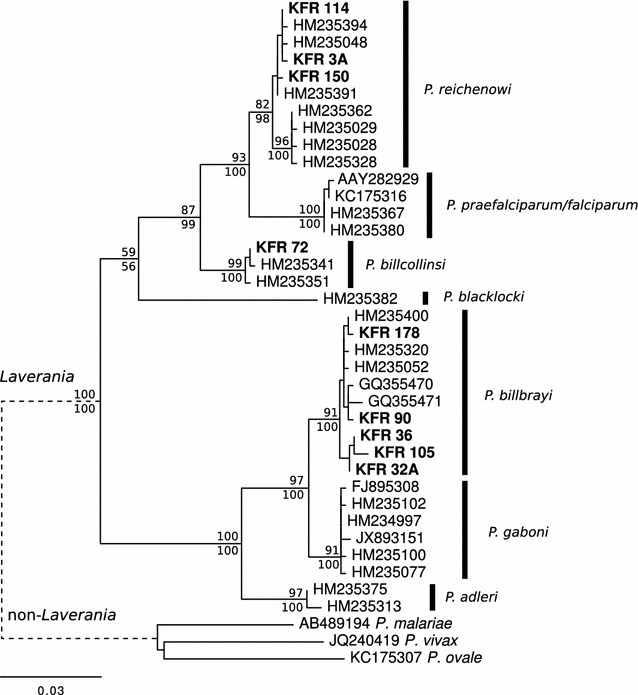
Phylogenetic tree of *Plasmodium* mitochondrial *cytochrome b* sequences (758 bp). Nodal support from 1000 bootstrap pseudoreplicates under ML and Bayesian methods are indicated above and below branches, respectively

In initial phylogenetic analysis, a geographical sub-structuring in *P. reichenowi* related to host phylogeography appeared (Fig. 3). A phylogram resulting from the extended dataset confirmed this sub-structure. All *P. reichenowi* sequences obtained from *P. t. schweinfurthii* formed a separated subclade as previously observed by Liu et al. [18]. This sub-structuring could be influenced by the geographical barriers or differences in mosquito vectors responsible for transmission of malaria parasites. Further investigation into ape-malaria from other chimpanzee populations, as well as the inclusion of environmental factors that may influence *Plasmodium* species distribution and abundance in wild great apes, will further contribute to a better understanding of *Plasmodium* species diversity and dynamics.

Of the two host traits analysed in this study, only age was found to be statistically significant, with young chimpanzees more likely to be infected with *Plasmodium* spp. than older ones. A similar trend was observed in western chimpanzees of Taï, Ivory Coast [14], western lowland gorillas inhabiting Dzanga-Sangha Protected Areas [15], as well as in humans [45, 46]. The time needed to develop semi-immunity against the malaria parasite may explain why *Plasmodium* was encountered more frequently among younger individuals [47]. Also the failure to find differences in infection levels between the sexes is consistent with previous results from western lowland gorillas [15] and western chimpanzees [14]. Indeed, the scarcity of information about the biology and ecology of *Laverania* lineages and their interactions with hosts, preclude us from drawing a precise picture of the infection dynamics.

The pattern of infections (negative-to-positive and vice versa) was observed in 18 individuals sampled more than once over the course of the sampling period. It is worth noting that negative samples observed in this study do not necessary reflect the absence of infections. Rather, this phenomenon might be explained by fluctuation of parasitaemia level and shedding of parasite DNA in faeces, combined with sensitivity of the *Plasmodium* detection in faecal samples expected to be lower compared to blood samples [18, 48]. These findings may indicate that detection of *Plasmodium* DNA in faeces is prone to high risk of false negativity, hindering adequate assessment of actual prevalence of malaria in free ranging chimpanzee populations.

## Conclusion

The findings of this study contribute to a broader understanding of malaria occurrence among wild chimpanzees. The differences observed may result from local variation in host exposure to mosquito vectors, extrinsic factors, differences in chimpanzee density, as well as host genetic related factors. Future research should focus not only on screening chimpanzees that live in a variety of habitats, but also identifying potential vectors and vector abundance, in order to provide insights on the distribution and occurrence of *Plasmodium* spp. in chimpanzees.

## Additional file

10.1186/s12936-016-1476-2 *Plasmodium* partial cytochrome b gene sequences obtained from GenBank and this study.
